# How personal values follow the societal lockdown due to COVID-19: Case of business students in Slovenia

**DOI:** 10.3389/fpsyg.2023.987715

**Published:** 2023-04-13

**Authors:** Vojko Potocan, Zlatko Nedelko

**Affiliations:** Department of Management and Organization, Faculty of Economics and Business, University of Maribor, Maribor, Slovenia

**Keywords:** personal values, COVID-19, societal lockdown, young adults (18-25 years old), changing values

## Abstract

We examined patterns of change and stability in four individual-level higher-order groups of Schwartz personal values among individuals during societal lockdown caused by COVID-19 epidemic. The study involves comparison of personal values of 85 business students during societal lockdown, with their personal values in pre- and post-pandemic periods. Sampled group of individuals includes undergraduate and graduate students from University of Maribor, Faculty of Economics and Business, Maribor, Slovenia. Contrary to previous publications, our results indicate a decrease of mean-level for all four higher-order groups of individuals’ values during societal lockdown of COVID-19. In the value hierarchy, self-transcendence values remain first, followed by conservation, openness to change, and self-enhancement values. In the period after the COVID-19, personal values again approached their pre-pandemic levels. Self-transcendence and conservation returned close to baseline levels, while openness to change and self-enhancement values exceeded initial pre-epidemic levels. In the value hierarchy, lead openness to change values, followed by the self-transcendence, self-enhancement, and conservation values. We discuss perceived changes in business students’ values due to the COVID-19 pandemic and present their capacities for dealing with potential unfavorable and threatening circumstances in the future.

## 1. Introduction

The outbreak of acute coronavirus syndrome - SARS-CoV-2 at the beginning of 2020 caused a global health pandemic to which governments worldwide have responded by different measures to contain the health crisis (Merkur et al., 2020; Stawicki et al., 2020). Introduced measures - from recommendations for health protection to total societal lockdowns, have worsened social circumstances and limited individuals’ personal and social lives (Haug et al., 2020; Wilder-Smith and Freedman, 2020). Countries are also faced with the need to ensure the necessary compliance and prosocial behavior of individuals needed to deal with COVID-19 (Kuper-Smith et al., 2020; Liekefett and Becker, 2021).

In framework of behavior we focus our intention on the state and changes of individuals’ values during the pandemic (Daniel et al., 2022) given that values decisively shape the full spectrum of human behavior (Rokeach, 1973; Inglehart and Baker, 2000).

Individuals’ values, their changes and consequently their behaviors explain theories of life stages (Robins et al., 2001) and generational cohorts (Meglino and Ravlin, 1998), because people probably adjust their values to their social circumstances, whether the circumstances are favorable (i.e., positive) or not [see Bardi et al. (2014), and Tormos et al. (2017)]. Empirical studies also evidence that individuals’ values orientations formed in childhood and youth remain relatively stable through their lives (Inglehart, 1997; Tormos et al., 2017) but they can also change due to radical changes in individuals’ live - e.g. Employment, family formation, illness, or radical changes in social circumstances for their lives (Inglehart, 1997; Tormos et al., 2017).

Thus, research reports on changes in human values during worsening of social circumstances in past societal crises (Bardi and Goodwin, 2011; Parks-Leduc et al., 2015). Previous crises leading to increase the importance of human values associated with surviving and protecting individual life (Parks-Leduc et al., 2015; Daniel et al., 2022) and reduces the importance of values associated with individual and social development (Mak et al., 2009; Park et al., 2017). Additionally, studies reported that altered personal values return to initial levels when social stability is restored (Inglehart and Baker, 2000; Schwartz et al., 2017). However, there is limited empirical evidence how development of individuals personal values follows changes of societal macro-level factors - such as society, culture, and economics, (Vecchione et al., 2016; Lam, 2021) and different phases of life stages (Schuster et al., 2019; Russo et al., 2022).

The issue of changing values in a crisis has become relevant again with the emergence of the COVID-19 epidemic, which has caused a unique health crisis characterized by global spread, longer duration, and strong deterioration of social circumstances (Stawicki et al., 2020; Wilder-Smith and Freedman, 2020). Among the measures taken to deal with the COVID-19 epidemic, the most widely shared opinion among researchers is the importance of introduced societal lockdowns - as measure which radically limited social life, to change personal values of individuals and different social groups in society (Park et al., 2017; Sortheix et al., 2019; Spahl et al., 2022).

That is why our conceptual goal is to study patterns of changes and stability of individuals’ values under the changing situational circumstances of COVID-19, as suggested several studies in the last decades (Tormos et al., 2017; Sortheix et al., 2019). Specifically, we investigated the changes of the personal values of sampled business students – as sample of young adults, during COVID-19 epidemic circumstances in the first half of 2020. Additional goal is to compare business students’ values under the COVID-19 societal lockdown with their values in the period before and after the societal lockdown.

Key rationales underlying this research are: circumstances of being and living are determine forming of individuals’ values especially in decisive stages of their moral and personal development (Russo et al., 2022); situational circumstances of major crises – as COVID-19, may lead to a changes of individuals’ values (Sortheix et al., 2019; Bojanowska et al., 2021; and present business students have never experienced such deterioration of societal living circumstances as caused with COVID-19 (Pavao, 2020; Grasso et al., 2021). Accordingly, we analyze changes of the patterns and hierarchy of personal values among individuals’ business students during the societal lockdown for manage of COVID-19 epidemic.

Students are in the decisive stage of moral and personal development (Tormos et al., 2017; Schuster et al., 2019; Russo et al., 2022), and the COVID-19 epidemic may trigger their stronger behavior responses compared to older individuals with more stable or permanent values. Analyze is aimed on the course of COVID-19 in Slovenia in the first half of 2020, which allows us to exclude from the analysis the specifics of COVID-19 among different countries and areas. Data was collected from a cohort of undergraduate and graduate students at the University of Maribor, Faculty of Economics and Business, who can be classified as social groups of young adults - according to The theory of life stages (Robins et al., 2001), and Generation Z - according to The theory of generational cohorts (Meglino and Ravlin, 1998). Analyzing a sampling cohort of business students enables consideration of personal values of individuals which share the similar personal values and values orientations (Meredith and Schewe, 1994).

The present study has both empirical and conceptual objectives to improve the behavior of sampled group of business students, and currently young adults in future societal crises. Our analysis contributes to the theory of personal values (Schwartz, 2012; Tormos et al., 2017) by exploring the relations between changing macro-level societal circumstances and the personal values of individuals (Frink et al., 2004; Mak et al., 2009). Next, we contribute to the development of a sociological understanding of young adults’ values (Parks-Leduc et al., 2015; Vecchione et al., 2016) by analyzing their actual changes in personal values in COVID-19 circumstances during the first half of 2020 (Wolf et al., 2020; Grasso et al., 2021). By differentiating business students from the entire population of young adults (Haski-Leventhal et al., 2017; Maloni et al., 2019), we want to expand the knowledge about their specific behaviors and boundary conditions what can help us understand their current and future behavior (Bardi et al., 2014; Park et al., 2017; Scholtz and Rennig, 2019).

In the following sections of this paper, we briefly describe the human values, and personal values of business’ students and young adults in general. Next, we present the COVID-19 epidemic as selected situational circumstances for our study and outline the reasons for this selection. Based on the selected theoretical framework, we continue with a theoretical discussion about the changing personal values of individuals during societal lockdown of COVID-19, which in turn leads to our development of research predicting about the changes of personal values of young adults during worsening and improving of societal circumstances from the perspective of value theories, situational theory, and health crisis management. We concluded the paper with a discussion of study results and possibilities of their use for improving young adults’ values/behavior in current and future societal crises.

## 2. Literature review and development of research

### 2.1. Literature review

#### 2.1.1. Human values

Literature presented several definitions of values among which is most often cited Meglino and Ravlin (1998) which define values “individuals’ beliefs about how they should behave in their social environment”, and Schwartz (1992) which explain that “personal values are abstract beliefs about desirable, trans-situational goals that serve as guiding principles in people’s lives”. Values considerably shape the full spectrum of human behavior (Schwartz, 1992; Russo et al., 2022) and many studies research the factors which influence and form the personal values (Parks-Leduc et al., 2015; Wilder-Smith and Freedman, 2020).

Our research follows studies of students and young adults’ personal values under crisis deterioration of societal circumstances (Rokeach, 1973; Inglehart and Baker, 2000; Schwartz et al., 2017; Maloni et al., 2019). In analyzing of individuals’ personal values, we aim on Schwartz’s Theory of basic human values developed (Schwartz, 1992) as predominant approach for consideration of values and psychology (Schuster et al., 2019; Vecchione et al., 2020; Russo et al., 2022).

Schwartz (1992) proposed the classification of all human values into ten broad groups of values - differentiated by their underlying goals of motivations, which he then combined into four higher-order groups of values in a circular structure. To deal with relationships among defined groups of values, the model was supplemented with a deal with two additional principles which can explain dynamic structure of values - namely (1) the interests that value attainment serves and (2) relations of values to anxiety, which can help in predicting and understanding relationships of values to various attitudes and behaviors. Schwartz (1992, 2012) also developed a consistent model for the consideration of values - named as “The quasi-circumflex model of values”.

Schwartz’s values model (Schwartz, 1992; Schwartz et al., 2012) has been widely used in the last decades to justify and evaluate the personal values of different populations, social groups, and cultures in over 80 countries (Egri et al., 2000; Reynaud et al., 2007; Schwartz et al., 2017; Spahl et al., 2022).

Due to changes in values and the development of people’s value orientations over time (Tormos et al., 2017; Spahl et al., 2022), past studies have not provided sufficient evidence for understanding of value orientation among current young adults from Generation Z. Therefore, we continue their consideration with analyzing of four higher-order groups of values - namely self-transcendence, self-enhancement, openness to change, and conservation, as defined by Schwartz (1992), Smith et al. (1996), and Ralston et al. (2011).

Self-transcendence indicates the extent to which one is motivated to promote the welfare of others and nature (universalism, benevolence). Self-enhancement indicates the extent to which one is motivated to promote self-interest, even when they are potentially at the expense of others (power, achievement, hedonism). Openness to change indicates the extent to which a person is motivated to follow his/her own intellectual and emotional interests in unpredictable and uncertain ways (stimulation, self-direction). Conservation indicates the extent to which one is motivated to preserve the status quo and the certainty that it provides in relationships with others, institutions and traditions (tradition, conformity, security) (Ralston et al., 2011). We followed Ralston et al. (2011) in creating four higher-order groups of values.

These groups of values help humans cope with one or more of the following universal requirements of existence: needs of individuals as biological organisms, requisites of coordinated social interaction and survival and welfare needs of groups (Schwartz, 2012) important in times of worsening environmental conditions.

#### 2.1.2. Behavior of business students

Main behavior theories define sampled business students as members of different social groups (Meredith and Schewe, 1994; Gilleard, 2004; Dimock, 2019). Sampled individuals according to developmental stage theories belong to young adults as a social group aged 18 to 25 years, in the phase following adolescence (Erikson, 1975; Levinson, 1986). According to Generation theory sampled students belongs to Generation Z (Newman and Newman, 2012; Dimock, 2019) as individuals born between the mid to late 1990s and early 2010s (Newman and Newman, 2012; Dimock, 2019; Alfirević et al., 2023). In Europe, young adults from Generation Z represent most of students’ populations, young adults comprise 13% of the active working population, while members of Generations Z represent more than 22% of the total EU population (Scholtz and Rennig, 2019; Gomez et al., 2020).

The behavioral characteristics of current business students reflect the general behavior of young adults from Generation Z which literature describes as digital individuals living and breathing through virtual connection, caring for the common good, and believing that everyone has the right to be what they want in their lives (Deal et al., 2010; Gomez et al., 2020; Alfirević et al., 2023). However, their comparison with past young adults’ behavior is difficult, since most of the past research was conducted with diverse samples of respondents (Tormos et al., 2017; Schuster et al., 2019; Leijen et al., 2022) and mainly in favorable societal and situational circumstances (Sortheix et al., 2019; Grasso et al., 2021).

We focused our research on examining a cohort of undergraduate and graduate students from one faculty in Slovenia as a social group of young adults that shares very similar life experiences from youth and are in the same phase of life and social development; thus, they are likely to have formed the similar value orientation (Bardi and Goodwin, 2011; Russo et al., 2022).

We used selected cohort for analyzing of their personal values and value orientations during social lockdown COVID-19 pandemic.

#### 2.1.3. Circumstances of the COVID-19 epidemic

The COVID-19 epidemic differs significantly from previous health-related societal crises mainly due to its global prevalence, recurring nature, large differences in the spread of disease between countries and areas worldwide, and diversified measures taken to manage it (Harper et al., 2020; Stawicki et al., 2020).

At the beginning of 2020, COVID-19 in Europe was marked by various circumstances from the 1st wave (from January to the end of June) through the summer period (from July to September) and to the 2nd wave (from September to the end of the year) of the epidemic (CRC, 2022; HSPM, 2022). The European countries responded to the outbreak of COVID-19 in early 2020 by activating and implementing various measures to manage the crisis, from preventive measures to complete restrictions on movement and social life in societies (Harper et al., 2020; Wilder-Smith and Freedman, 2020; Casquilho-Martins and Belchior-Rocha, 2022).

Different used approaches for managing the COVID-19 health crisis in Europe also open the question about meaning of different epidemic measures for changing of personal values among individuals in Europe (Haug et al., 2020; Grasso et al., 2021; Spahl et al., 2022). That is why we focused our research on the situational circumstances during the 1st wave of the epidemic (CRC, 2022; HSPM, 2022) and researched pandemic circumstances in Slovenia, as case of European country that introduced one of the most restrictive societal lockdown to manage the COVID-19 (GCO, 2022; NIPH, 2022).

The 1st wave of COVID-19 in Slovenia was marked by: (1) a pre-epidemic period from the beginning of 2020 to the first national confirmed case on March 04, (2) a period from declaring an epidemic on March 04 to May 15 when Slovenia declared the end of the COVID-19 epidemic as the first country in Europe, and (3) post-epidemic period from May 15 to end of June 2020, when the European Union officially declared the end of the 1st wave of epidemic in Europe (GCO, 2022; NIPH, 2022). Our government introduced very restrictive and generally valid social lockdowns of the entire society in the country to contain the epidemic, which completely stopped the entire social life in the country for the first time after the epidemic of smallpox in 1972 (GCO, 2022; HSPM, 2022).

### 2.2. Development of research

#### 2.2.1. Personal values of business students

The discussed business students (Haski-Leventhal et al., 2017; Potocan and Nedelko, 2021; Nedelko et al., 2022) – as well as other current young adults in Europe (Scholtz and Rennig, 2019; Spahl et al., 2022), grew up in conditions of prosperity, constant progress and social-economic security. Predominantly favorable conditions in their childhood and growing up were led formation of the similar personal values (Vecchione et al., 2016; Tormos et al., 2017; Nedelko et al., 2022).

Such conditions lead to the development of post-modernistic values related to self-confidence, skepticism, and care for responsible relations with others (Meglino and Ravlin, 1998; Parks-Leduc et al., 2015; Schwartz et al., 2017) and shape their individualistic values orientation. Schwartz’s values typology (Schwartz, 2012) supports increased openness to change and self-transcendence values and decreased importance of modernistic survival values. Social researchers presume that circumstances of societal security have a weak direct effect on the gradual development of personal values in the short term, while in the long term, such circumstances affect values indirectly through shared life experiences of individuals (Meglino and Ravlin, 1998; Tormos et al., 2017). Social scholars also agree that the same circumstances equally affect the personal values of individuals and social groups with similar values orientations (Bardi et al., 2009; Schwartz et al., 2017).

Several empirical studies report similar developmental trends of values and value orientations among younger adults (Bardi et al., 2014; Schwartz et al., 2017). Thus, the research of Sortheix et al. (2019) reports that similar social security conditions lead to the development of similar value orientations of individuals. Vecchione et al. (2020) find in their research that favorable social circumstances increase the importance of openness to change and values of self-transcendence among adults in Europe.

However, the question remains open, whether past crises in Europe - especially a global economic crisis in 2009 and the refugee crisis (Sortheix et al., 2019; Chmel et al., 2021) and their deterioration of social circumstances in the childhood of considered business students in Slovenia (Potocan and Nedelko, 2021; Nedelko et al., 2022), and young adults in Europe in general (Vecchione et al., 2016; Tormos et al., 2017) influenced the formation of personal values of individuals needed to face the COVID-19 crisis. Research on this issue so far indicates that previous crises did not significantly impact on the development of personal values of currently young adults, especially because of weaker impact and short duration of these crises in Europe (Scholtz and Rennig, 2019; Sortheix et al., 2019).

Growing up of young adults in prevailing conditions of prosperity and social security open questions about their ability to cope with the radical deterioration of living circumstances (Vecchione et al., 2016; Wilder-Smith and Freedman, 2020), and their willingness to change their personal values in worsening circumstances (Grasso et al., 2021; Casquilho-Martins and Belchior-Rocha, 2022).

The introduction of social lockdowns and other measures in Slovenia and Europe to manage COVID-19 restricted social life of societies and each individual (CRC, 2022; HSPM, 2022), especially the young generation in Europe (Chmel et al., 2021; Passini, 2022). Young adults are for the first time in their lives faced with life-threatening circumstances and previously unknown obstacles (Wolf et al., 2020; Spahl et al., 2022). The introduced COVID-19 measures were limited their social development especially their gain desired social status, the transition from school to work, and establishing in the labor market (Liekefett and Becker, 2021; Daniel et al., 2022; Mohanty and Sharma, 2022). In that context, it is worth examining how the introduction of societal lockdowns caused by COVID-19 changed the personal values of business students and young adults in general (Kuper-Smith et al., 2020; Grasso et al., 2021).

According to values, past crises indicate that societal insecurity results in an increase of young adults social-focused values – as responsibility and conservation (Schwartz, 1992; Sortheix et al., 2019) and values focused to control of people and material resources (Parks-Leduc et al., 2015; Vecchione et al., 2016). At the same time, the deterioration of societal circumstances results in a decrease of their values associated with avoiding conflicts, unpredictability, and compliance with societal expectations (Frink et al., 2004; Schwartz et al., 2012).

#### 2.2.2. Stability of the personal values

Inglehart (1997) grounded the starting point for understanding the stability of individuals’ personal values under changing circumstances, indicating that personal values are relatively stable motivational characteristics, but can be changed in response to major changes in personal or social circumstances (Robins et al., 2001; Bardi et al., 2009; Tormos et al., 2017). Later researches supplemented Inglehart’s work (1997) with findings on the duration and strength of changed personal values - i.e. their stability, under changing circumstances (Bardi and Goodwin, 2011; Harper et al., 2020; Russo et al., 2022).

Research so far has revealed basic circumstances and mechanisms behind the value change (Rokeach, 1973; Inglehart, 1997; Bardi et al., 2014), and possible approaches or viewpoints for consideration of values stability – such as rank-order stability, mean-level change, stability in within values hierarchies, and patterns of intra- values changes. More about stability of values see in Schwartz (1992), Bardi and Goodwin (2011), Schwartz (2012), and Vecchione et al. (2016). Among the possible approaches for research of issues related with stability vs. change of values, we focused our attention on the analysis of changes in the achieved levels of values for sampled individuals and selected period of time.

Past research indicates a different connection between the stability of personal values and specific societal circumstances (Bardi and Goodwin, 2011; Tormos et al., 2017). Studies have reported that favorable social circumstances characterized by social security do not lead to change of individuals’ values immediately, but may indirectly change their personal values through life experiences of individuals over time (Robins et al., 2001; Schwartz et al., 2017; Sortheix et al., 2019). In contrast, authors from social sciences have found that worsening social circumstances lead to the immediate change in values, which is mainly temporary and returns close to the baseline level after the normalization of circumstances (Mak et al., 2009; Haug et al., 2020; Vecchione et al., 2020). In addition, some newest research has revealed that confronting individuals with unknown dangers and intense threats to their existence can lead to large and longitudinal changes in their values (Sortheix et al., 2019; Daniel et al., 2022).

Past researches regarding the stability of personal values of individuals also revealed several contextual and methodological gaps, such as differences between uncertainty in society and individual perception of (actual and perceived) level of uncertainty, realization that actual uncertainty in society does not always increase people’s awareness of their uncertainty and that the individuals awareness of uncertainty does not always lead to an actual change in his/her value orientations (Inglehart and Baker, 2000; Schwartz et al., 2017).

The theoretical and empirical works about the short-term duration of personal values changes during previous crises in Europe (Inglehart and Baker, 2000; Daniel et al., 2022), return of altered personal values to initial levels when social stability is restored (Inglehart and Baker, 2000; Schwartz et al., 2017) substantiated our prediction that social circumstances in period after COVID-19 epidemic will lead to the immediate changes of young adult values.

Based on these arguments, we continue with research of personal values of business students before, during and after the COVID-19’ social lockdown.

## 3. Materials and methods

### 3.1. Instrument

We used a questionnaire entitled “A survey of work-related issues,” developed by The University Fellows International Research Consortium (University of Oklahoma)” under the supervision of Prof. dr. David Ralston and his research group (Egri and Ralston, 2004; Furrer et al., 2010) to collect data from business students. The questionnaire consists of four parts. The first part includes a list of personal values defined in Schwartz’s value survey (Schwartz, 1992). The second part includes 38 short scenarios (Egri et al., 2000) about possible workplace advancement strategies. The third part consists of 25 items that measure the economic, natural, and social aspects of an organization’s corporate social responsibility (Furrer et al., 2010). The last part contains ten personal and organizational demographic variables typically used in business studies (Egri and Ralston, 2004). The questionnaire was initially developed for surveying employees (Potocan and Nedelko, 2021); therefore, we adapted the wording for surveying the student population in Slovenia and beyond (Nedelko et al., 2022). With student questionnaires, we collected data annually since 2006. In 2020, besides the annual collection, we additionally collected responses from a cohort of business students to observe more closely the impact of COVID-19 circumstances on business students.

### 3.2. Sample and procedure

The survey was conducted in the academic year 2019/2020 among business students pooled from the bachelor’s and master’s level management program at the Faculty of Economics and Business, University of Maribor, Slovenia. Our convenience sample comprised a cohort (Meredith and Schewe, 1994; Gilleard, 2004) of 85 business students surveyed three times in the first half of 2020. The survey was conducted for the first time before the epidemic, from 13. 01. to 24. 01. 2020 (*N* = 85) The second repetition was conducted after the lockdown due to the COVID-19 was officially declared in Slovenia, from 07.04. to 17. 04. 2020 (*N* = 85). The third repetition was done after the lockdown was officially cancelled, from 25. 05. to 05. 06. 2020 (*N* = 85). We didn’t consider survey repetitions as related samples, as we need to ensure anonymity of participants, and we did not relate each participant’s answer during three observations. We considered each repetition as entity of 85 individuals on aggregate level.

The average age of respondents was 22.61 years at the time of the first survey wave. In sample were 37.6 percent males and 62.4 percent females. In terms of level of study, 38.8 percent were bachelor students and 61.2 percent master students. We followed ethical procedures when conducting the survey and work with data, as well as we ensured the anonymity of the respondents in line with good ethical practices of research work. All students participated voluntarily and did not receive any reward for participation in the survey.

We collected responses in the following way. We created an online survey, where all fields were set as mandatory to prevent the submission of incomplete surveys. We sent the link to the online survey to the selected participants, instructing them to take a screenshot after completing the survey, which indicate that the survey was submitted successfully. They were also instructed to cut out the system date in the right corner of the screenshot and hide the device IP (if it appears in the screenshot) and send it to the principal investigator. Thus, the principal investigator can keep the record who submitted the questionnaire, but he cannot link individual’s response in the survey database with his/her name, as the time, date of submission and IP from respondent’s device is not seen. Thus, we were able to keep the evidence who submitted the survey, while also keeping respondent’s anonymity. The principal investigator sent reminder one week after the survey opened in each of three surveying period.

### 3.3. Measures

Personal values were measured using the Schwartz Value Survey (SVS) (Schwartz, 1992), in which the respondents rated the importance of 56 single values on a 9-point interval scale, ranging from “opposed to my values” (-1) to “of supreme importance” (7). For this study we followed the practice of using 45 items (Egri and Ralston, 2004; Ralston et al., 2011) with the aim to achieve satisfactory reliability of newly formed constructs (Schwartz, 1992). We collapsed 45 single values into four higher-order groups of values, namely self-transcendence (*N* = 13; α = 0.87), self-enhancement (*N* = 10; α = 0.90), openness to change (*N* = 8; α = 0.88), and conservation (*N* = 14; α = 0.87), which were used in this study. The obtained reliabilities were well above the commonly accepted threshold of 0.7 (Nunnally, 1978) and greater than the previously reported Cronbach’s alpha coefficients for self-transcendence (α = 0.82), self-enhancement (α = 0.78), openness to change (α = 0.74), and conservation (α = 0.80) in the study of employees across 48 societies (Ralston et al., 2011).

To examine the importance of personal values in different circumstances, we distinguished between (1) period before the lockdown, which characterizes “the normal situation in society” as it was known before the advent of COVID-19, (2) period of the lockdown, referring to the time of closure of society due to the measures to contain the epidemic of COVID 19, and (3) period after the lockdown when the measures to curb the epidemic of COVID 19 were lifted. Accordingly, we created a set of two dummy variables, setting the period before the lockdown as a reference category.

As controls, we used the respondents’ self-reported (1) age, (2) gender, and (3) undergraduate or graduate level of study.

### 3.4. Research design and analyses

Our research followed three steps. First, we outlined the elements of the descriptive statistics, including mean values, standard deviation, median, as well as skewness and kurtosis. The absolute values of skewness and kurtosis for four higher-order groups of values, that are in center of our research (see Table 1), are less than 1.0, which indicate on slight non-normality (Lei and Lomax, 2005). Additionally, we checked for outlines, using boxplots, and we can conclude that there are no outliners in the data. Thus, we proceed, with parametric tests. With use of the analysis of variance (ANOVA), which is considered “robust” (George and Mallery, 2019, p. 222), the impact on the results is negligible and is in line with prevalent practice of using parametric test in surveying personal values in business research (Ralston et al., 2011).

**TABLE 1:** Descriptive statistics for study variables^a^.

Variable	Mean	Std. deviation	Median	Skewness	Kurtosis
(1) Self-transcendence	4.56	0.98	4.69	−0.55	0.11
(2) Self-enhancement	3.90	1.32	4.20	−0.72	0.07
(3) Openness to change	4.22	1.21	4.50	−0.74	0.17
(4) Conservation	4.00	1.05	4.21	−0.59	0.01

^a^*N* = 255.

Second, we outlined the importance of four groups of values in observed period and examined differences in the importance of personal values of business students before the societal lockdown, during the lockdown, and after the lockdown, by using ANOVA (Table 2). We also report multiple comparisons between three considered periods (before lockdown vs. lockdown, before lockdown vs. after lockdown, lockdown vs. after lockdown), to determine differences in the importance of personal values between each considered pair (Table 3).

**TABLE 2:** Individual level higher-order dimensions of personal values before the lockdown, during the lockdown, and after the lockdown.

Variables	Before lockdown		Lockdown		After lockdown		
	Mean	SD	Mean	SD	Mean	SD	*F*
Self-transcendence	4.64	1.27	4.40	0.96	4.64	0.58	1.83
Self-enhancement	4.10	1.43	3.30	1.40	4.29	0.83	14.91***
Openness to change	4.42	1.42	3.53	1.16	4.71	0.54	26.40***
Conservation	4.23	1.19	3.58	1.10	4.20	0.67	11.17***

^a^***p < 0.001.

**TABLE 3:** Mean differences and post hoc results for individual-level higher-order dimensions of personal values before the lockdown, during the lockdown, and after the lockdown^a^.

A (status)	B (status)	Mean difference (A-B)			
		Self-transcendence	Self-enhancement	Openness to change	Conservation
Before lockdown	Lockdown	0.25	0.80***	0.89***	0.65***
	After lockdown	0.00	−0.19	−0.29	0.04
Lockdown	Before lockdown	−0.25	−0.80***	−0.89***	−0.65***
	After lockdown	−0.25	−0.99***	−1.18***	−0.62**
After lockdown	Before lockdown	0.00	0.19	0.29	−0.04
	Lockdown	0.25	0.99***	1.18***	0.62**

^a^Mean difference is significant at **p < 0.01; ***p < 0.001.

Third, we followed procedure established by Potocan and Nedelko (2021), and examined the associations between the considered periods, i.e., before the societal lockdown, during the lockdown, and after the lockdown, and the importance of personal values. We examined whether for instance if the impact before lockdown significantly different from the impact of lockdown. Here we followed suggestions of Cumming (2009). Using his “inference by eye approach,” where 95 per cent confidence intervals are observed, we assess whether there is significant difference between compared values of correlation coefficients. When 95 per cent confidence intervals do not overlap, two tailed *p* value is less than 0.001, and when two confidence intervals just touch, two tailed value *p* is less than 0.01. Intervals can overlap as much as about half the length of the one confidence internal arm before *p* becomes as large as 0.05. In all those instances, there is statistically significant difference between the compared values of correlation (Table 4). In frame of bivariate correlation analysis, we performed bootstrapping, where correlations between three periods and four groups of values were calculated (Table 5).

**TABLE 4:** Correlations between circumstances before the lockdown, during the lockdown, and after the lockdown and individual level higher-order dimensions of personal values^a^.

Dependent variable	Before lockdown			Lockdown			After lockdown		
	95% confidence interval			95% confidence interval			95% confidence interval		
	Standardized coefficient (β)	Lower bound	Upper bound	Standardized coefficient (β)	Lower bound	Upper bound	Standardized coefficient (β)	Lower bound	Upper bound
Self-transcendence	0.06	−0.08	0.21	−0.12	−0.24	−0.01	0.06	−0.04	0.16
Self-enhancement	0.11	−0.02	0.23	−0.32***	−0.43	−0.20	0.21**	0.12	0.30
Openness to change	0.12	−0.03	0.25	−0.41***	−0.51	−0.30	0.29***	0.20	0.38
Conservation	0.16*	0.03	0.28	−0.29***	−0.39	−0.17	0.13*	0.03	0.23

^a^*N* = 255; *p < 0.05; **p < 0.01; ***p < 0.001.

**TABLE 5:** The differences in correlations between circumstances before the lockdown, during the lockdown, and after the lockdown and individual level higher-order dimensions of personal values.

Variable	Before lockdown vs. lockdown	Before lockdown vs. after lockdown	Lockdown vs. after lockdown
Self-transcendence	*p* < 0.05*	*p* > 0.05	*p* < 0.01**
Self-enhancement	*p* < 0.001***	*p* > 0.05	*p* < 0.001***
Openness to change	*p* < 0.001***	*p* < 0.05*	*p* < 0.001***
Conservation	*p* < 0.001***	*p* > 0.05	*p* < 0.001***

***The difference between standardized beta coefficients for the considered pair was statistically significant at *p* < 0.001.

**The difference between standardized beta coefficients for the considered pair was statistically significant at *p* < 0.01.

*The difference between standardized beta coefficients for the considered pair was statistically significant at *p* < 0.05.

All the calculations were done in IBM SPSS 27.0.

## 4. Results

Descriptive statistics for variables of interest for the aggregated sample is presented in Table 1.

The results for aggregated sample of business students reveal that most important individual-level higher-order groups of personal values are self-transcendence values, followed by openness to change and conservation, while self-enhancement values are least important. Next, we are examining the importance of individual-level higher-order groups of personal values before, during, and after the lockdown, as well as revealing difference in the importance of personal values in different observed points (Table 2). Plots for four groups of values are outlined in Supplementary Appendix 1.

Table 3 summarizes multiple comparisons between four groups of individual higher order groups of value during three periods considered.

Tables 2, 3 reveal that period of the societal lockdown is characterized by substantial decrease of all considered groups of values. The mentioned tables reveal that during the period of societal lockdown the importance of self-enhancement and conservation values of business students, compared to the period before the COVID-19, is decreased. During the societal lockdown also, the importance of openness to change and self-transcendence (not significant decrease) values of business students, compared to the period before the COVID-19, is decreased.

The period after the COVID-19 epidemic increased the importance of self-transcendence (not significant increase) and openness to change personal values for business students compared to the period of the COVID-19 epidemic. After lockdown circumstances also increased the importance of conservation and self-enhancement personal values of business students compared to the period of the COVID-19 epidemic.

Finally, we examined how important are considered periods, i.e., before the societal lockdown, during the lockdown, and after the lockdown, for changing of considered groups of values. In Table 4 we present correlations between circumstances before lockdown, lockdown, and after lockdown and individual level higher-order groups of personal values. Table 5 summarizes the impact of three considered circumstances on each of the individual-level higher order groups of values, following suggestions of Cumming (2009).

Table 4 reveals a statistically significant impact of lockdown on lowering the importance of groups of personal values, with the exception of self-transcendence values. There is also a moderate statistically significant influence of the post-lockdown period on the values of openness to change and self-enhancement, as their importance rises above the pre-lockdown importance.

The differences in correlations between circumstances before the lockdown, during the lockdown, and after the lockdown and individual level higher-order groups of personal values are presented in Table 5.

The results in Table 5 show that the impact of societal lockdown on all four groups of values was statistically different from the impact of circumstances the before lockdown on values. Additionally, there is also statistically significant difference in the impact of societal lockdown and the after lockdown period on all value groups. Finally, comparing the impact of the pre- and post-lockdown periods on values revealed a statistically significant impact of the post-lockdown period compared to the pre-epidemic period only for openness to change values.

## 5. Discussion

This study examined differences in the importance of personal values among a cohort of business students in Slovenia before, during, and after the COVID-19 epidemic, by focusing on the first wave of COVID-19 in the first half of 2020.

The epidemic radically worsened the European population’s life circumstances and especially complicated the social development of business students and young adults in Slovenia and around Europe (Harper et al., 2020; Wolf et al., 2020). In accordance with previous cognitions about terrorist attacks (Verkasalo et al., 2006), wars (Daniel et al., 2013; Sundberg, 2016), economic recession (Park et al., 2017; Sortheix et al., 2019), and outbreak of diseases (Frink et al., 2004; Mak et al., 2009; Nel Van Zyl et al., 2021) circumstances of a greater threat to people’s health and un-security of their lives increase their pro-social personal values. In contrast, these societal circumstances decrease people’s motivation to realize their interests and consider the welfare of others and nature (Inglehart and Baker, 2000; Parks-Leduc et al., 2015).

Results of our research shows that during the societal lockdown decreased the importance of business students’ higher-order groups of personal values, whereby the openness to change and self-enhancement values decreased more, and conservation and self-transcendence values decreased less (Bardi and Goodwin, 2011). The study thus confirms the theoretical presumptions about decreasing of students’ motivation to realize their interests and consider the welfare of others and nature during the crisis (Inglehart and Baker, 2000; Parks-Leduc et al., 2015), but does not confirm the presumptions about increasing their pro -social personal values (Frink et al., 2004; Mak et al., 2009) during the societal lockdown of COVID-19. The additional state of personal values of students during the crisis follows the presumptions of previous studies that people during societal and health insecurity of various crises value more the importance of self-protection values and less self-expansive values (Inglehart, 1997; Bardi et al., 2009).

In retrospect, a general decrease in values may make sense because young adults were confronted with the health threat and total lockdown of societal activities for the first time in their lives facing severe societal life restrictions and unexpected loss of relatives (Dimock, 2019; Pavao, 2020). Considering the period after the social lockdown, the importance of values returned to the initial level or above initial level, confirming a temporary change of values importance due to the worsening social circumstances (Mak et al., 2009; Haug et al., 2020; Vecchione et al., 2020). This probably indicate existence of the immediate and short-term impact of COVID-19 epidemic measures on the values of young adults. The high volatility of values due to crisis can also be attributed to the fact that young adults have less stable values than adults (Sundberg, 2016; Vecchione et al., 2016).

Our observations revealed association between lockdown circumstances during COVID-19 epidemic and the change in the hierarchy of personal values of business students. Considering the hierarchy of values of young adults, our cohort of business students valued self-transcendence value the most. Similar results can be found in other studies of Generation Z, where self-transcendence values were at the forefront, emphasizing universalism and benevolence that define this group of values (Bojanowska et al., 2021; Nedelko et al., 2022). Thus, the surveyed individuals attached more importance to the values related to their individual development, self-expansion in society, and care for the common good (Scholtz and Rennig, 2019; Vecchione et al., 2020), consistent with the past research on the values of Generation Z (Scholtz and Rennig, 2019; Pavao, 2020). Students supported self-protection and anxiety-avoidance values less due to relative security while growing up (Inglehart and Baker, 2000; Schwartz, 2012). Such a pattern of respondents’ values follows the circular value structure proposed by Schwartz (1992) and is in line with past research on Generation Z’s value development (Dimock, 2019; Scholtz and Rennig, 2019).

During the epidemic, individuals highlighted the importance of values related to protecting individuals’ well-being, while previous studies have reported mainly the importance of conservation and self-enhancement values in worsening circumstances (Frink et al., 2004; Mak et al., 2009). Respondents attached less importance to openness to change and presentable self-enhancement values, which the previous studies highlighted as the key factors motivating individuals’ compliance and engagement in prosocial behavior in crises (Bardi et al., 2014; Sortheix et al., 2019).

Research proves that self-transcendence values are most stable, as their importance remains high and does not change substantial (nor significantly) due to the changed circumstances. At the same time, the importance of other values decreased significantly during the crisis. Our results contradict the previous studies, which reported an increase in conservation and self-enhancement values and a decrease in openness to change and self-transcendence values in times of crisis (Frink et al., 2004; Bardi et al., 2014). Openness to change is the most volatile value and susceptible to crises, as supported by a previous study indicating that the importance of openness to change diminishes in a crisis (Sortheix et al., 2019; Bojanowska et al., 2021).

Due to the societal lockdown during COVID-19 epidemic, we would most likely expect the strengthening of values related to conservatism, which highlights security, conformity, and tradition. This group of values strengthened during the crisis but did not push self-transcendence values out of the first place. The conservation value was not expected to rise (Vecchione et al., 2020), which can be attributed to the increased concern for security in society and the measures to contain the epidemic, i.e., conformity values. This can be due to the impact of the crisis and its specificity, which put health and society at the forefront. After the crisis, conservation returned to the last place in the hierarchy of values, indicating a temporary change of conservation values importance due to the worsening social circumstances (Mak et al., 2009; Haug et al., 2020; Vecchione et al., 2020).

In addition to the evident influence of societal lockdown on changes in the importance of values, a comparison of the importance of values before and after societal lockdown revealed that self-transcendence and conservation values have almost returned to their initial level, while the values of self-enhancement, and especially open to change, have exceeded the original level, which contradicts the expect return to the baseline, when circumstances normalize (Mak et al., 2009; Haug et al., 2020; Vecchione et al., 2020). As the possibility of achieving goals related to excitement, novelty, and independence was limited due to the social lockdown, it is somehow logical that the importance of these values increased while the importance of the values associated with “social lockdown” diminished the most. These considerable shifts in importance can also be attributed to the longevity of measures, especially from the point of view of young adults who have a reduced ability to pay constant attention and want to resolve issues as quickly as possible (Dimock, 2019) to “catch up.”

The substantial rise in importance of openness to change can also be attributed to the life stage of development, which is characterized by an increase in the importance of the values of self-transcendence (Vecchione et al., 2020), as young adults prefer the excitement, novelty, the challenge in life, independent thought and action, and pleasure.

Especially interesting are the results about values of business students in the period after the COVID-19 social lockdown, because business students will have a significant influence on shaping the functioning and behavior of organizations, as well as entire society indirectly in the future. From a content point of view, the state of personal values in the post-crisis period needs to be considered on the basis of a comparison with the state of values before the crisis as achieved levels of development of personal values of students. Such an approach theoretically based on assumptions about the relative stability of individual values (Inglehart, 1997; Tormos et al., 2017) and that changed values in crisis altered to initial level when social stability is restored (Inglehart and Baker, 2000; Schwartz et al., 2017).

Our results show that in the period after the crisis values openness to change and self-enhancement values recorded the largest increase. The values of openness to change, what can be attributed to the student’s desire to return to normal social life, as a fundamental advantage of ending the epidemic, increased the most. The increase in the level of self-enhancement can be partially explained by the normalization of life in society, which has enabled students to again achieve their professional and social empowerment aimed at increasing power and achievement, as well as partially by hedonism, and emphasizes pursuit of one’s own interests and relative success.

On the contrary, the importance of conservation values has decreased, which indicates the tiredness of students with restrictions during the time of COVID-19 and the desire of students to act more freely and live with less orders, self-restriction, and preservation of the past. In comparison with the period before the crisis, only self-transcendence values remained unchanged, as these are to the greatest extent defined by the dominant behavioral orientation of students in universalisms and benevolence as typical characteristics young adults and especially Generation Z (Nedelko et al., 2022; Alfirević et al., 2023).

According to hierarchy of personal values, on the first place is openness to change, followed by self-transcendence, self-enhancement, and conservation. The jump of openness to change to first place in the hierarchy of values in the post-lockdown period may be attributed to the fact that “for the first time in their lives, students were forced into social isolation and faced with very restrictive life restrictions” during society lockdown, and that is why they so highly rated the importance of re-opening of the society for returning to “normal life”. This is followed by the values of self-enhancement, which are ranked first in most of the crisis situation reviews (Frink et al., 2004; Mak et al., 2009), but our study ranks them second. The exchange of places of those groups of values in the hierarchy can be partially explained by the behavioral response of the students, who estimate that the opening of society is the most important condition for their further implementation and is therefore more important than self-transcendence values. The values of self-enhancement and conservation also changed places in the hierarchy of students’ values. We can partly explain such a change with the possibility of students reasserting themselves in the environment, what encourages their desire and the possibility of emphasizing the pursuit of one’s own interests and relative success and dominance over others. Partly, such hierarchy also reflects the boredom of students with the obligation to comply with the rules of social lockdown, which were not always sufficiently embodied, not presented objectively enough in the public, and were not compared to milder COVID-19 measures in other environments.

To summarize, current young adults are in the decisive stage of moral and personal development (Bardi et al., 2014; Tormos et al., 2017) and COVID-19 epidemics triggered stronger behavior responses in this particular cohort compared to older individuals with more permanently formed values (Bardi et al., 2009; Parks-Leduc et al., 2015; Sundberg, 2016).

### 5.1. Implications

This seminal study of short-term changes in values due to crisis circumstances has several theoretical and practical implications. It covered three significantly different periods, pre-epidemic, epidemic and post-epidemic, during the first half of 2020.

Previous studies have monitored the value change due to the global financial crisis for more than a decade (Tormos et al., 2017; Sortheix et al., 2019) or a shorter period of 10 months (Bojanowska et al., 2021). We used a single cohort to rule out inter-generational and inter-cultural differences (Meredith and Schewe, 1994; Gilleard, 2004). Our breakthrough theoretical contribution is that the importance of values in crisis, in our case, societal lockdown due to COVID-19, is also reduced in the short term, although values are traditionally considered stable (Bardi et al., 2014; Vecchione et al., 2020). This research contributes to the theory of personal values (Schwartz, 2012; Tormos et al., 2017) by exploring the relations between changing macro-level societal circumstances and the personal values of individuals (Frink et al., 2004; Mak et al., 2009). We empirically verified and contributed to the literature by showing that situational changes have an initial strong impact on the importance of individuals’ values in the short term (Frink et al., 2004). Further, we found out that values are strengthened again after the crisis and return close to or even exceed the pre-epidemic importance. Most stable values among studied business students are self-transcendence, while the most volatile value is openness to change. These findings contribute substantially to the value theory and the impact of the crisis on short-term value changing and help us understand their current and future behavior (Bardi et al., 2014; Park et al., 2017; Scholtz and Rennig, 2019).

This study’s most notable practical implication is analyzing actual changes in young adults’ personal values in COVID-19 circumstances during the first half of 2020 (Wolf et al., 2020; Grasso et al., 2021) and emphasizing that societal lockdown has significantly lowered the importance of openness to change, self-enhancement, conservation values, and to a lesser extent, self-transcendence values. Therefore, the diminishing importance of business students’ values due to the crisis circumstances (i.e. societal lockdown) is extremely important in light of the current situation, when we have a war in Ukraine, the looming financial and economic crisis, and a general negative outlook on further economic growth. We need to account for substantial short-term impact of these situational circumstances on personal values and their hierarchy (Daniel et al., 2013; Sundberg, 2016; Vecchione et al., 2020). More attention should be given to moral development and increasing the awareness of the importance of values among current young adults, as these protracted events could significantly reduce the importance of values and lead to a crisis of values in the long run. In addition, young adults are concerned about their studies, learning, summer/part-time jobs, and internships (Pavao, 2020), which could further undermine the importance of values in this group.

Within the individual level, openness to change values as a higher-order dimension of values is most likely to decrease in times of change. Those values support creativity (Lebedeva et al., 2019), which is important for the development of society. Therefore, it is important to pay enough attention to these values, as economic activity is declining due to the crisis. High volatility of openness to change values among younger generations requires more attention (Merkur et al., 2020; Wolf et al., 2020), as young adults are important drivers of economic and social progress, especially business students, who will significantly influence the functioning and behavior of organizations in the future, and consequently also entire society. This suggests that the repeated occurrence of crises would significantly affect the functioning of entities in society and that major societal events will diminish the importance of openness to change (Daniel et al., 2013; Bojanowska et al., 2021. Therefore, competent institutions must draw up action plans to mitigate the lowering of those values when dealing with future crises.

### 5.2. Limitations

This study has the following limitations. The central constraint is that the focus was on one social cohort of business students from one cultural cluster belonging to young adults from Generation Z to avoid possible divergence in the values (Tormos et al., 2017; Nedelko et al., 2022; Alfirević et al., 2023) and the importance and hierarchy of values between generations (Bardi et al., 2014; Vecchione et al., 2016). This limits the generalization of the results to the whole population of young adults and older generations, although the key insights could also be applied to current population of young adults, with appropriate critical judgment. The perceived impact of societal lockdown on the importance of values might be biased, as young adults’ values are less stable than those of adults (Sundberg, 2016; Vecchione et al., 2016) and are more prone to rapid change in difficult situations (Daniel et al., 2013; Passini, 2022). It is also necessary to acknowledge that the change in values may be due to young adults’ current life development phase (Vecchione et al., 2020), which was not controlled in the study due to the short time interval of the study. We focused on individual-level higher-order groups of values, while other levels (Schwartz, 2012) and other classifications of personal values were not considered. A limitation also represents smaller number of individuals involved in the research and the predominance of female in the sample. As another limitation can be outlined also using parametric tests in our analysis, despite some deviation from theoretical normal distribution exists in our data. But, testing differences in mean values based on the results of Kruskal-Wallis test, allows exactly the similar conclusions as with the tests we used in our study.

### 5.3. Future research directions

Our study is unique and cannot be replicated due to the specificity of the epidemic and the inability to retrospectively study the impact of societal lockdown in other cultural circles or among other generations. The most promising avenue is to examine the changes in personal values orientations and the development of personal values and hierarchy of values in the further course of the COVID 19 epidemic not covered in this survey (Haug et al., 2020; Wolf et al., 2020), preferably in the same generation of young adults. This would clarify the long-term impact and strength of the impact of the COVID-19 phenomenon and related measures on the importance of values for young adults, whose values are most susceptible to change (Daniel et al., 2013; Vecchione et al., 2016). Furthermore, it would explain whether the substantial fall in the importance of personal values is due to the first wave of the COVID-19 epidemic or whether it also occurred in the following stages of the epidemic with the introduction of epidemic measures (Haug et al., 2020; Wilder-Smith and Freedman, 2020). Such a study would elucidate the impact of strong social challenges on changes in the importance of values and their hierarchy, especially if we consider current re-occurring events, such as the financial and economic crisis. It would be interesting to explore the development of young adults’ values through the lens of individuals vs. collectivism due to the growing importance of individualism for young adults.

## Data availability statement

The raw data supporting the conclusions of this article will be made available by the authors, without undue reservation.

## Ethics statement

The studies involving human participants were reviewed and approved by the ethics committee at the Faculty of Economics and Business (University of Maribor). The participants provided their written informed consent to participate in this study.

## Author contributions

Both authors listed have made a substantial, direct, and intellectual contribution to the work, and approved it for publication.
