# Supplementary material for: How personal values follow the societal lockdown due to COVID-19: Case of business students in Slovenia
Source: Front Psychol. 2023 Apr 13;14:987715. doi: 10.3389/fpsyg.2023.987715 (PMC10140780; doi:10.3389/fpsyg.2023.987715)

Appendix 1

Self-enhancement values before, during, and after the lockdown

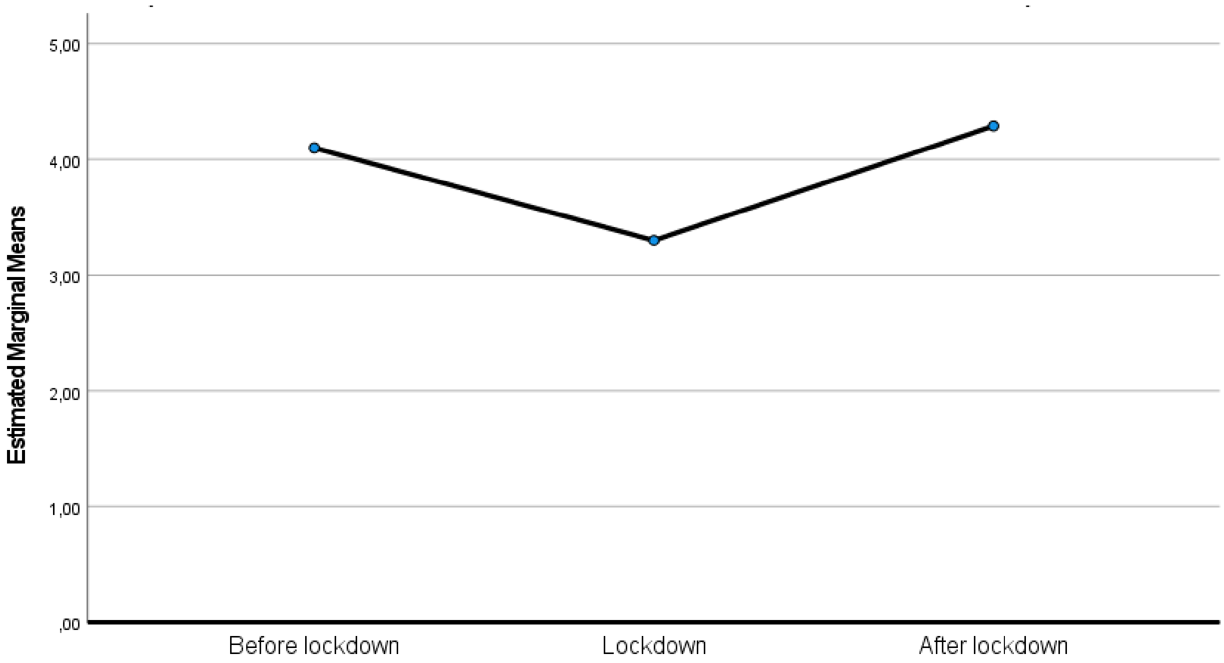

Self-transcendence values before, during, and after the lockdown

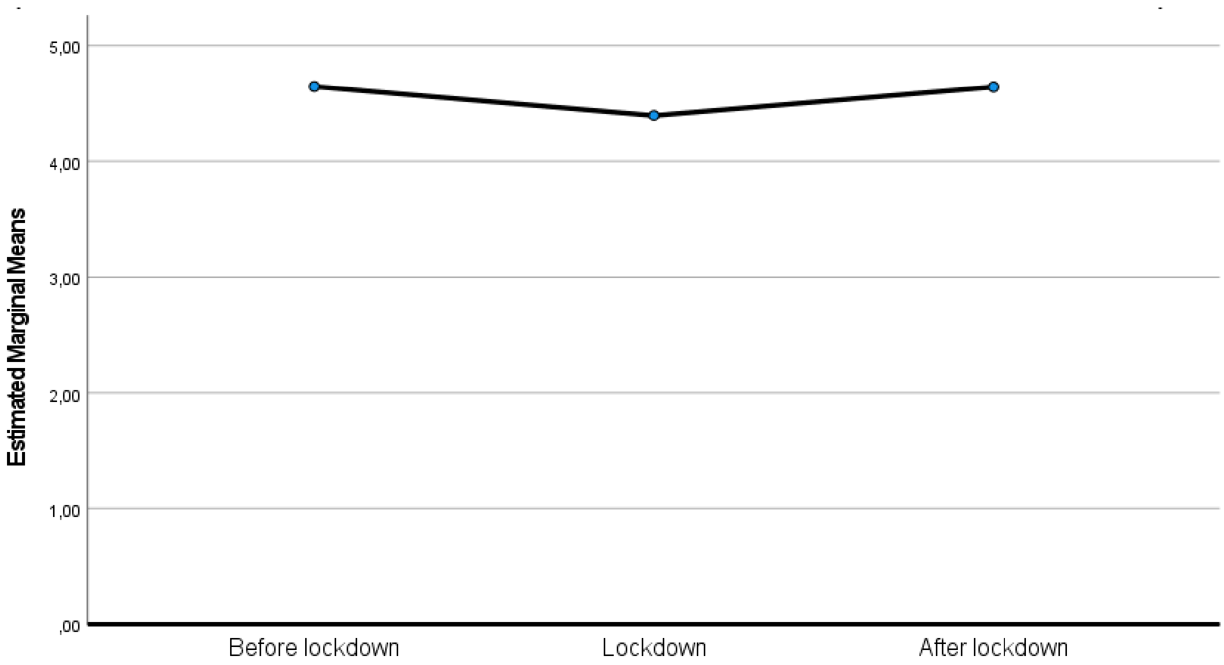

Openness to change values before, during, and after the lockdown

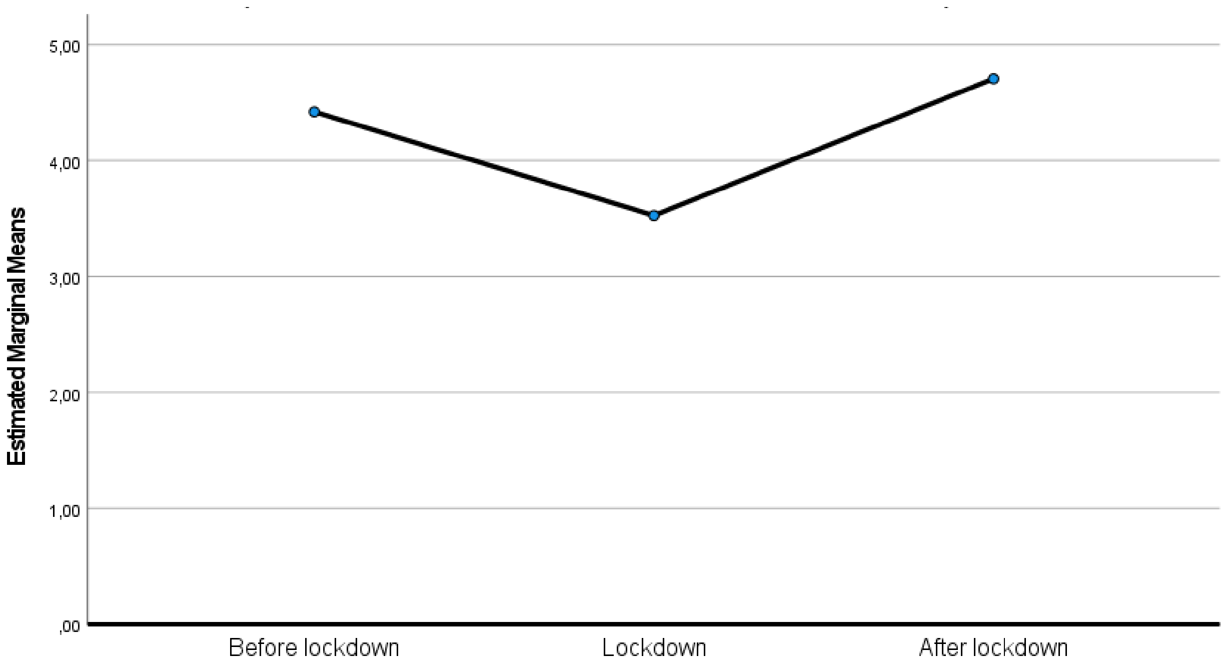

Conservation values before, during, and after the lockdown

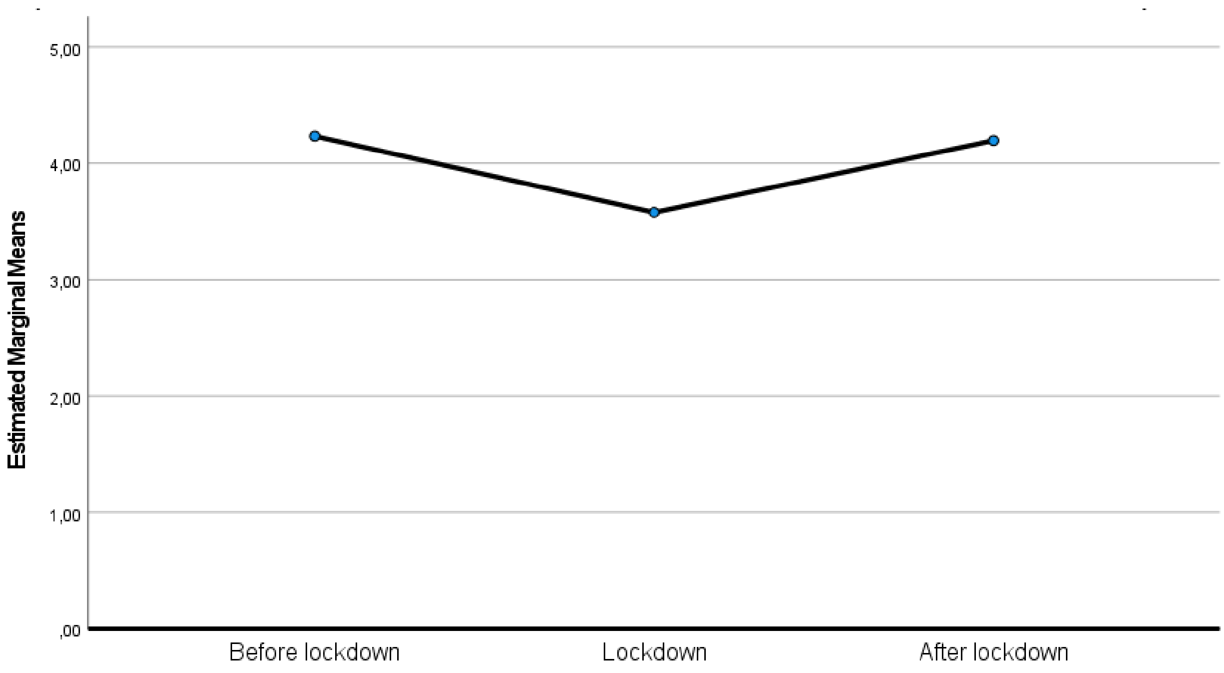

Individual-level higher-order groups of personal values before, during, and after the lockdown

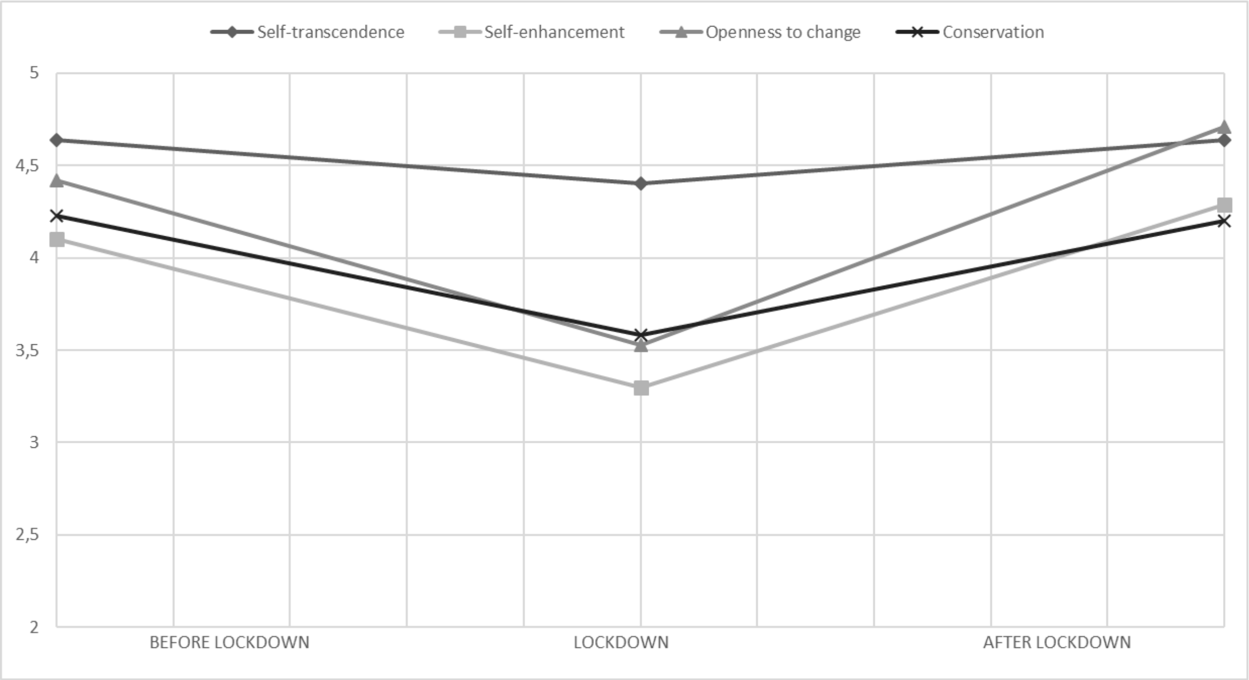

Supplement: Supplementary file 1 [file Data_Sheet_1.pdf]
